# Early vascular deficits are correlated with delayed mammary tumorigenesis in the MMTV-PyMT transgenic mouse following genetic ablation of the NG2 proteoglycan

**DOI:** 10.1186/bcr3174

**Published:** 2012-04-24

**Authors:** Krissa Gibby, Weon-Kyoo You, Kuniko Kadoya, Hildur Helgadottir, Lawrence JT Young, Lesley G Ellies, Yunchao Chang, Robert D Cardiff, William B Stallcup

**Affiliations:** 1 Cancer Center, Tumor Microenvironment Program, Sanford-Burnham Medical Research Institute, 10901 North Torrey Pines Road, La Jolla, CA 92037 USA; 2 Center for Comparative Medicine, University of California at Davis, County Road 98 and Hutchison Drive, Davis, CA 95616 USA; 3 Department of Pathology, University of California at San Diego, 9500 Gilman Drive, UC303 Room 102, La Jolla, CA 92093-0063 USA; 4 Department of Research Evaluation and Scientific Programs, Susan G. Komen for the Cure, 5005 LBJ Freeway Suite 250, Dallas, TX 75244 USA; 5 Histology Group, Viacyte Inc., Bldg 2 Room 305, 3550 General Atomics Court, San Diego, CA 92121 USA; 6 Medical Direction Department, Biolumina, 75 Varick Street 10th Floor, New York, NY 10013 USA; 7 Department of Biochemistry, St. Jude Childrens's Research Hospital, 262 Danny Thomas Place, Memphis, TN 38105 USA

## Abstract

**Introduction:**

The neuron-glial antigen 2 (NG2) proteoglycan promotes pericyte recruitment and mediates pericyte interaction with endothelial cells. In the absence of NG2, blood vessel development is negatively impacted in several pathological models. Our goal in this study was to determine the effect of NG2 ablation on the early development and function of blood vessels in mammary tumors in the mammary tumor virus-driven polyoma middle T (MMTV-PyMT) transgenic mouse, and to correlate these vascular changes with alterations in mammary tumor growth.

**Methods:**

Three different tumor paradigms (spontaneous tumors, transplanted tumors, and orthotopic allografts of tumor cell lines) were used to investigate the effects of NG2 ablation on breast cancer progression in the MMTV-PyMT transgenic mouse. In addition to examining effects of NG2 ablation on mammary tumor growth, we also investigated effects on the structure and function of tumor vasculature.

**Results:**

Ablation of NG2 led to reduced early progression of spontaneous, transplanted, and orthotopic allograft mammary tumors. NG2 was not expressed by the mammary tumor cells themselves, but instead was found on three components of the tumor stroma. Microvascular pericytes, myeloid cells, and adipocytes were NG2-positive in both mouse and human mammary tumor stroma. The effect of NG2 on tumor progression therefore must be stromal in nature. Ablation of NG2 had several negative effects on early development of the mammary tumor vasculature. In the absence of NG2, pericyte ensheathment of endothelial cells was reduced, along with reduced pericyte maturation, reduced sprouting of endothelial cells, reduced assembly of the vascular basal lamina, and reduced tumor vessel diameter. These early deficits in vessel structure are accompanied by increased vessel leakiness, increased tumor hypoxia, and decreased tumor growth. NG2 ablation also diminishes the number of tumor-associated and TEK tyrosine kinase endothelial (Tie2) expressing macrophages in mammary tumors, providing another possible mechanism for reducing tumor vascularization and growth.

**Conclusions:**

These results emphasize the importance of NG2 in mediating pericyte/endothelial cell communication that is required for proper vessel maturation and function. In the absence of normal pericyte/endothelial cell interaction, poor vascular function results in diminished early progression of mammary tumors.

## Introduction

In addition to factors intrinsic to tumor cells, elements of the tumor microenvironment also have profound influences on mammary tumor progression and metastasis [1-3]. Even when mammary epithelial cells are transformed by oncogenes, such as mouse mammary tumor virus-driven polyoma middle T (MMTV-PyMT) [4], stromal/epigenetic factors are required for progression from hyperplasias to malignancies [5,6]. Key mammary tumor stromal elements include the vasculature, adipocytes, fibroblasts, myeloid cells, and the extracellular matrix, as well as matrix-associated proteases and growth factors. Of these stromal components, the vasculature is the most universal and widely recognized [7]. The transition of hyperplastic foci to neoplasms requires the recruitment of a vascular supply, an event known as the angiogenic switch [7,8]. Effective tumor vascularization is critical not only for growth of the primary neoplasm, but also for tumor metastasis to distant sites.

We have shown that the NG2 proteoglycan (also known as CSPG4, AN2, HMPG, and HMW-MAA) is a prominent cell surface component of microvascular pericytes and can serve as a reliable marker for detection of these cells during the early stages of microvessel development [9-12]. In addition, because NG2 is important for cell proliferation, motility, and cell-cell interaction [13], genetic ablation of NG2 [14] results in deficient vascularization in several models of postnatal angiogenesis. In both ischemic retinal vascularization and corneal angiogenesis, the NG2 null mouse exhibits reduced microvessel formation due to retarded pericyte function. In these models, deficits in both pericyte recruitment and proliferation lead to reduced pericyte investment of endothelial cells [15]. In an allograft model of brain tumor progression, ablation of NG2 causes a several-fold reduction in tumor progression due to deficits in pericyte/endothelial cell interactions that lead to poor vascular function. In particular, the reduced ensheathment of endothelial cells by NG2 null pericytes causes deficiencies in basal lamina assembly, vessel patency, and vessel integrity that compromise vessel performance [16]. These results emphasize the functional importance of NG2 in stimulating pericyte proliferation and motility, possibly via NG2-mediated enhancement of pericyte responses to growth factors such as FGF2 [10,12,13], as well as the role of NG2 in mediating β1 integrin activation that promotes pericyte/endothelial cell interaction during early phases of neovascularization [17].

The MMTV-PyMT transgenic mouse provides a means of studying the stromal role of NG2 in a model of spontaneous breast cancer initiation and progression. Although in some cases of human basal-like breast cancer NG2 is reportedly expressed by tumor cells that are triple-negative for estrogen receptor, progesterone receptor, and HER2 [18,19], we have found that NG2 is not expressed by mammary tumor cells in the MMTV-PyMT mouse [11,20]. Thus, in the transgenic mouse model, any effects of NG2 ablation on mammary tumor progression must be due to alterations in stromal influences. The MMTV-PyMT mouse is ideal for this work for several reasons. First, mammary tumors occur in 100% of female MMTV-PyMT mice [21]. Second, tumor onset is rapid compared to most other mammary tumor models [22]. Third, the model exhibits many features of human breast cancer [4]. Fourth, the fact that NG2 is not expressed by the mammary tumor cells in this model allows us to focus on the stromal, as opposed to tumor cell-autonomous, roles of the proteoglycan. This pattern of expression is relevant to human breast cancer, where NG2 is also strongly expressed by tumor stromal elements. Stromal roles of NG2 are equally relevant to NG2-positive and NG2-negative mammary tumor types. Here we demonstrate that ablation of NG2 in the MMTV-PyMT mouse causes vascular deficits during the early stages of tumor development and that these deficits correlate with a reduction in early mammary tumor growth.

## Materials and methods

### Animals

Wild type (NG2+/+) and NG2 null (NG2-/-) females [14] were crossed with males carrying the mouse mammary tumor virus promoter-driven polyoma middle T transgene (Tg) (MMTV-PyMT) [21] to generate C57Bl/6 wild type and NG2 null versions of this breast cancer model [23]. β-actin/EGFP transgenic mice (C57Bl/6; Jackson Laboratories, Bar Harbor, ME, USA) were maintained on both wild type and NG2 null backgrounds. Mice were housed in the Sanford-Burnham Vivarium (fully accredited by the Association for Assessment and Accreditation of Laboratory Animal Care). All animal procedures were performed in accordance with Office of Laboratory Animal Welfare regulations and were approved by Sanford-Burnham Institutional Animal Care and Use Committee review prior to execution.

### Human tissues

Samples of human ductal adenocarcinoma were obtained from the Sanford-Burnham tumor bank. Surgical samples were obtained with the consent of the patients, and were taken prior to initiation of any treatment. Specimens were fixed in formalin at the time of surgical resection.

### Tumor cell lines

The Py230 and Py8119 mammary tumor cells lines were established from spontaneous mammary tumors arising in C57Bl/6 MMTV-PyMT females. Tumors were minced and rotated at 250 rpm at 37^°^C for three hours in 3 ml of Ham's F12K medium containing 1 mg/ml collagenase (Worthington Biochemicals, Lakewood, NJ, USA), 2 mg/ml soybean trypsin inhibitor (Sigma-Aldrich, St. Louis, MO, USA), and 2% BSA. After addition of FCS-containing medium, the suspension was passed through a 70 μm nylon filter (Fisher Scientific, Pittsburg, PA, USA). Single cells were pelleted by centrifugation and cultured in Ham's F12K medium containing 5% FCS, 2.5 μg/ml amphotericin B, 50 μg/ml gentamycin, and MITO+. Cell lines were cloned by limiting dilution in this same medium.

### Spontaneous tumor progression

For monitoring the progression of spontaneous MMTV-PyMT neoplasms, #4 mammary glands were spread as whole mounts on glass slides and stained with carmine-alum [6,24] to visualize the mammary epithelium, lymph nodes, mammary intraepithelial neoplasias (MINs), and incipient tumors. Early neoplastic progression (weeks 6 to 12) was quantified by using image analysis to determine the total area occupied by MINs. This analysis of MIN area did not include sub-areolar tumors, which were the earliest neoplasms to develop in both wild type and NG2 null MMTV-PyMT females. During weeks 14 to 20, the total mammary tumor burden was determined by weighing tumors dissected from all mammary glands.

### Tumor transplantation/engraftment

For mammary tumor transplantation, tumor fragments (1 mm^3^) were prepared from a single tumor (1 cm in diameter) taken from a wild type MMTV-PyMT donor, and transplanted bilaterally into the #4 fat pads of wild type and NG2 null recipient females. Tumor sites were palpated three times per week to determine the initial time at which tumors could be detected.

Engraftment of mammary tumor cell lines was accomplished by injection of Py230 and Py8119 cells (10^6 ^cells in 50 μl of PBS) into the #2 and #4 mammary fat pads of wild type and NG2 null mice. Tumor sites were palpated three times weekly to determine the initial time at which tumors could be detected.

### Immunostaining and microscopy

Dissected tumors or tumor-containing tissues were fixed in 1% paraformaldehyde and cryoprotected by overnight immersion in 20% sucrose. Tissue blocks were frozen in Optimal Cutting Temperature embedding medium (OCT, Sakura Finetek, Torrance, CA, USA) and used to prepare 20 μm sections on a Reichert-Jung 1800 cryostat microtome. Immunofluorescence was performed essentially as described [9] using the following primary antibodies: rabbit and guinea pig anti-NG2 [9,25], rat anti-mouse CD31, rat anti-mouse F4/80, and rat anti-mouse CD11b (BD Biosciences, La Jolla, CA, USA), mouse anti-α-smooth muscle actin (αSMA, Sigma-Aldrich), rabbit anti-desmin (Millipore, Billerica, MA, USA), rabbit anti-collagen IV (Millipore), and goat antibodies against VEGF and VEGFR-3 (R&D Systems, Minneapolis, MN, USA). Images were acquired using the Fluoview 1000 Laser Point Scanning Confocal Microscope (Olympus, Tokyo, Japan) and the Radiance 2100 Multiphoton Confocal Microscope (BioRad, Hercules, CA, USA). Additional fluorescence imaging was performed with the TE300 Nikon Fluorescence Microscope (Nikon, Tokyo, Japan). Image analysis was performed using Image-Pro Plus 4.5 software (Media Cybernetics Inc, Bethesda, MD, USA).

### Bone marrow transplantation

Bone marrow transplantation from female β-actin/EGFP donors to female MMTV-PyMT recipients was carried out as previously described [12]. Bone marrow was collected from tibiae and femurs of both wild type and NG2 null donors. Recipients at eight weeks of age (both wild type and NG2 null MMTV-PyMT females) were gamma irradiated with two doses of 5 Gy each, administered three hours apart, and were immediately reconstituted by retro-orbital injection of 5 × 10^5 ^bone marrow cells in 100 μl of Ringer's solution. Wild type bone marrow was transplanted to both wild type and NG2 null recipients, and a parallel procedure was performed with NG2 null bone marrow. Engrafted mice were maintained on antibiotic water (1.1 g/L neomycin sulfate and 455 mg/l Polymyxin B) for six weeks. Peripheral blood samples were collected from 14-week old mice for flow cytometric analysis of the extent of EGFP engraftment. Animals with at least 75% engraftment were utilized for analysis of macrophage populations as described below. In C57Bl/6 MMTV-PyMT mice, 14 weeks is roughly the earliest time at which mammary tumors can be detected by palpation [23]. Tumors were collected for flow cytometric analysis at ages ranging from 16 to 18 weeks.

### Flow cytometry

Spontaneous MMTV-PyMT mammary tumors (between 1 to 2 cm^3 ^in volume) were used for flow cytometric analysis of myeloid cell populations derived from β-actin/EGFP bone marrow donors. Tumors were taken from four sets of mice: wild type recipients engrafted with wild type bone marrow, wild type recipients engrafted with NG2 null bone marrow, NG2 null recipients engrafted with wild type bone marrow, and NG2 null recipients engrafted with NG2 null bone marrow. Tumor-bearing mice under Avertin anesthesia were perfused transcardially with 10 ml of cold saline. Tumors were dissected free of normal tissue, finely minced with a razor blade in (D)MEM/F12 medium, and digested with 2 mg/ml collagenase for one hour at 37°C. Digests were filtered through 70 μm nylon mesh to produce single cell suspensions, which were treated on ice for 30 minutes with Live/Dead Aqua (Invitrogen, Carlsbad, CA, USA) to allow exclusion of dead cells from flow cytometric analysis. Cells were then washed in PBS, pelleted at 1000 × g, and resuspended at a density of 10^5 ^cells in 100 μl PBS. These cell suspensions were treated on ice for 30 minutes with the following fluorochrome-labeled antibodies: CD11b APC, 1/100 dilution (eBioscience; San Diego, CA, USA); F4/80 PE-Cy5, 1/20 dilution (Biolegend; San Diego, CA, USA); CD45 PE-Cy7, 1/50 dilution (eBioscience); Gr1 APC-Cy7, 1/100 (Biolegend); Tie2 PE, 1/50 dilution (eBioscience); CD206 Alexa Fluor 647, 1/20 dilution (Biolegend); and CD11c Cy7, 1/50 dilution (eBioscience).

Labeled cells were pelleted at 1000 × g, resuspended for five minutes at room temperature in 100 μl of 1% paraformaldehyde, and then brought to 400 μl with PBS. Fluorescence activated cell sorting (FACS) was performed using the LSR Fortessa instrument (BD Biosciences; La Jolla, CA, USA). FloJo software (Tree Star, Inc; Ashland OR, USA) was used for quantitative analysis of the flow cytometric data.

### Vessel leakiness and tumor hypoxia

Leakiness of mammary tumor vessels and tumor hypoxia were determined after intravenous injection of fluorescein isothiocyanate (FITC)-dextran (250 kDa; Sigma-Aldrich) or pimonidazole hydrochloride (HPI Inc, Burlington, MA, USA), respectively [16].

### Statistical analysis

Quantitative results are expressed as means ± SE. Most statistical analyses were performed using two-tailed t-tests. Wilcoxon signed rank tests were used to evaluate statistical significance in the case of tumor onset studies. *P *values less than 0.05 were considered statistically significant.

## Results

### NG2 expression in mammary tumor stroma

**Figure 1:**
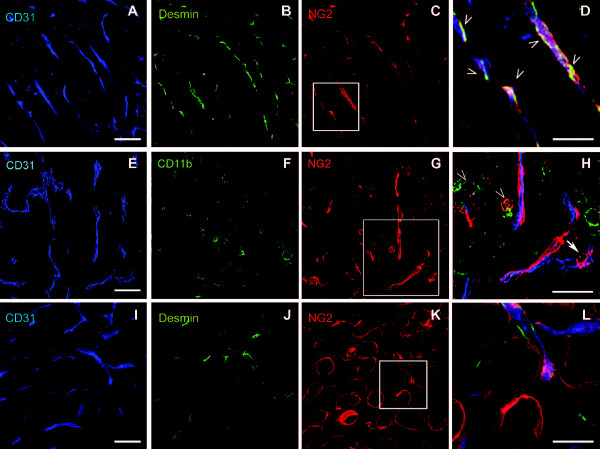
**NG2 expression in mouse mammary tumor stroma**. Sections of 16-week MMTV-PyMT mammary tumors were immunolabeled to demonstrate NG2 expression by elements of the tumor stroma. (**A-D**) Sections were labeled for CD31 (blue), desmin (green), and NG2 (red) to demonstrate the co-expression of desmin and NG2 by pericytes in close association with microvascular endothelial cells. D: high magnification of the boxed area in C. Arrowheads indicate pericytes expressing both desmin and NG2. (**E-H**) Sections were labeled for CD31 (blue), CD11b (green), and NG2 (red) to demonstrate the expression of NG2 by a subpopulation of CD11b-positive macrophages. H: high magnification of the boxed area in G. Arrowheads indicate macrophages positive for both CD11b and NG2. Arrow denotes blood vessel-associated CD11b-positive, NG2-positive macrophage. (**I-L**) Sections containing both normal and tumor tissue were labeled for CD31 (blue), desmin (green), and NG2 (red) to demonstrate the non-vascular expression of NG2 by adipocytes in the mammary fat pad. L: high magnification of the boxed area in K. Scale bars = 25 μm in D, H, and L. In all other panels, scale bars = 50 μm. MMTV-PyMT, mammary tumor virus-driven polyoma middle T; NG2, nerve-glial antigen 2.

An understanding of the role of NG2 in mammary tumorigenesis requires a knowledge of the cell types that express NG2. In sections of mammary tumors from 16-week old MMTV-PyMT mice, NG2 expression is seen on desmin-positive pericytes that are closely associated with CD31-positive endothelial cells in the tumor microvasculature (Figure 1A-D). As noted previously, NG2 is not detected on the mammary tumor cells themselves. NG2 is also expressed by a subpopulation of myeloid cells in MMTV-PyMT tumors. Tumor sections from 16-week old MMTV-PyMT mice contain CD11b-positive macrophages, some of which are positive for NG2 (Figure 1E-H). Some of these macrophages are closely associated with tumor blood vessels (arrow in H). Immunolabeling of sections of 16-week old MMTV-PyMT mammary tissue also reveals strong NG2 expression by adipocytes in the mammary fat pad (Figure 1I-L).

**Figure 2:**
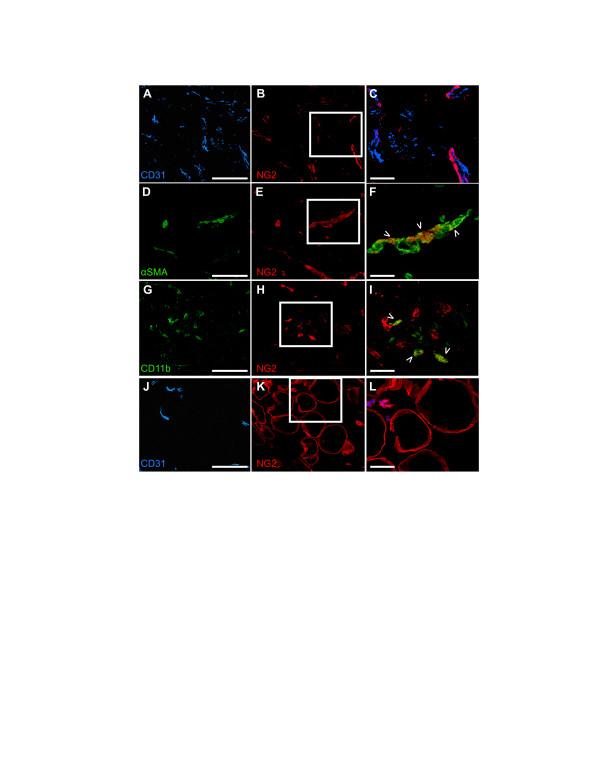
**NG2 expression in human mammary tumor stroma**. Sections of non-triple negative human ductal adendocarcinoma were immunolabeled to demonstrate NG2 expression by elements of the tumor stroma. (**A-C**) Sections were labeled for CD31 (A, blue) and NG2 (B, red). C, merged images from boxed area in B, showing close apposition of pericytes and endothelial cells. (**D-F**) Sections were labeled for αSMA (D, green) and NG2 (E, red). F, merged images from boxed area in E. Arrowheads indicate overlap between NG2 and αSMA labeling. (**G-I**) Sections were labeled for CD11b (G, green) and NG2 (H, red). I, merged images from boxed area in H. Arrowheads indicate overlap between NG2 and CD11b labeling. (**J-L**) Sections were labeled for CD31 (J, blue) and NG2 (K, red). L, merged images from boxed area in K). Images shown are representative of six ductal adenocarcinoma specimens that we examined. Scale bars = 25 μm in C, F, I, L. In all other panels, scale bars = 100 μm. Specimens 1, 3, and 8 in Table 1 were used to obtain these images. NG2, nerve-glial antigen 2; αSMA, α smooth muscle actin.

To determine if this pattern of stromal NG2 expression also occurs in human mammary tumors, we examined samples from a panel of 11 non-triple negative human ductal adenocarcinomas (Table 1). In the majority of these specimens (8 of 11), NG2 is not expressed by the mammary tumor cells, similar to the case of the MMTV-PyMT tumors. However, NG2 is found on many αSMA-positive pericytes (Figure 2D-F) that associate tightly with CD31-positive vascular endothelial cells (Figure 2A-C; Table 1). NG2 is also present on a population of CD11b-positive myeloid cells (Figure 2G-I; Table 1) and on adipocytes associated with the mammary gland (Figure 2J-L; Table 1). These labeling patterns are seen in tumors expressing various combinations of HER2, estrogen receptor, and progesterone receptor (Table 1), suggesting that stromal NG2 expression is relatively independent of the receptor profile of the tumor. Tumor cell expression of NG2 also did not appear to exhibit any clear association with receptor profile. The similarity of the human NG2 stromal expression pattern to that seen in MMTV-PyMT tumors establishes the relevance of our mouse work to human breast cancer.

**Table 1:** NG2 expression in human ductal adenocarcinoma.

Receptor Expression					NG2 expression			
**Sample**	**Stage**	**HER2**	**ER**	**PR**	**tumor cells**	**pericytes**	**macrophages**	**adipocytes**

1	2	6+	4+	-	-	3+	ND	4+
2	3	-	4+	-	2+	2+	ND	2+
3	3	-	4+	3+	-	2+	ND	2+
4	2	-	-	3+	-	1+	ND	ND
5	2	-	4+	-	-	2+	1+	ND
6	2	ND	4+	3+	2+	1+	ND	2+
7	2	2+	4+	3+	-	2+	ND	ND
8	1	ND	4+	3+	-	1+	1+	1+
9	1	2+	4+	3+	-	2+	1+	ND
10	1	3+	4+	3+	2+	2+	ND	2+
11	2	-	4+	ND	-	2+	ND	2+

Intensity of receptor or NG2 expression was judged by immunofluorescence on a scale of 1 to 6, with 6 being the most intense. Pathological tumor stages are graded on a scale of 1 to 4, with 4 being the most malignant. NG2 expression in pericytes was judged by triple staining for NG2, α-smooth muscle actin, and CD31. NG2 expression in macrophages was judged by double staining for NG2 and CD11b. Adipocytes were recognized on the basis of size and morphology (some samples did not have recognizable adipocytes, hence the ND notation). Images in Figure 2 were taken from samples 1, 3, and 8. ER, estrogen receptor; HER2, HER2 EGF receptor; ND, not determined; PR, progesterone receptor.

### NG2 ablation has little effect on normal mammary gland morphogenesis

**Figure 3:**
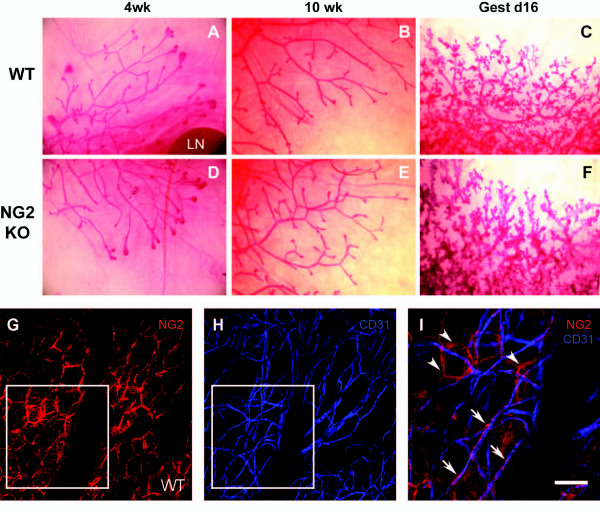
**Mammary gland development in wild type and NG2 null mice**. Whole mounts of #4 mammary glands from female wild type (WT: **A, B, C**) and NG2 null (NG2 KO: **D, E, F**) mice at the ages of four weeks, ten weeks, and 16 days of gestation (Gest 16d) were stained with carmine alum to visualize development of the mammary epithelium [24]. No changes in mammary gland expansion or branching were noted in the NG2 null mouse. LN: lymph node. (**G-I**). Whole mounts of normal #4 mammary gland from a 12-week old wild type female were immunostained for NG2 (G, red) and CD31 (H, blue). Panel I shows merged images from the boxed area in G and H. NG2 expression is seen on adipocytes (arrowheads) and on pericytes (arrows) that are closely apposed to CD31-positive endothelial cells. Scale bar = 60 μm (G, H) and 30 μm (I). NG2, nerve-glial antigen 2.

Since aberrant development of the mammary epithelium could affect mammary tumor progression, it was necessary for us to compare mammary gland morphogenesis in wild type and NG2 null mice. Using whole mounts of #4 mammary glands, we determined that the extent of mammary gland expansion and the ductal branching pattern of the mammary gland at four and ten weeks of age are not detectably altered in the NG2 null mouse (Figure 3A-F). Similarly, the dramatic expansion and morphogenesis of ducts during pregnancy are not noticeably affected by NG2 ablation. Figure 3G-I demonstrates that NG2 expression in normal mammary gland is associated with blood vessels (arrows) and adipocytes (arrowheads), just as seen in the case of mammary tumor stroma (Figures 1 and 2). Assessment of NG2 expression by macrophages in normal mammary tissue was hindered by the scarcity of resident myeloid cells under non-pathological conditions.

### Effect of NG2 ablation on progression of MMTV-PyMT tumors

#### Progression of spontaneous mammary tumors

**Figure 4:**
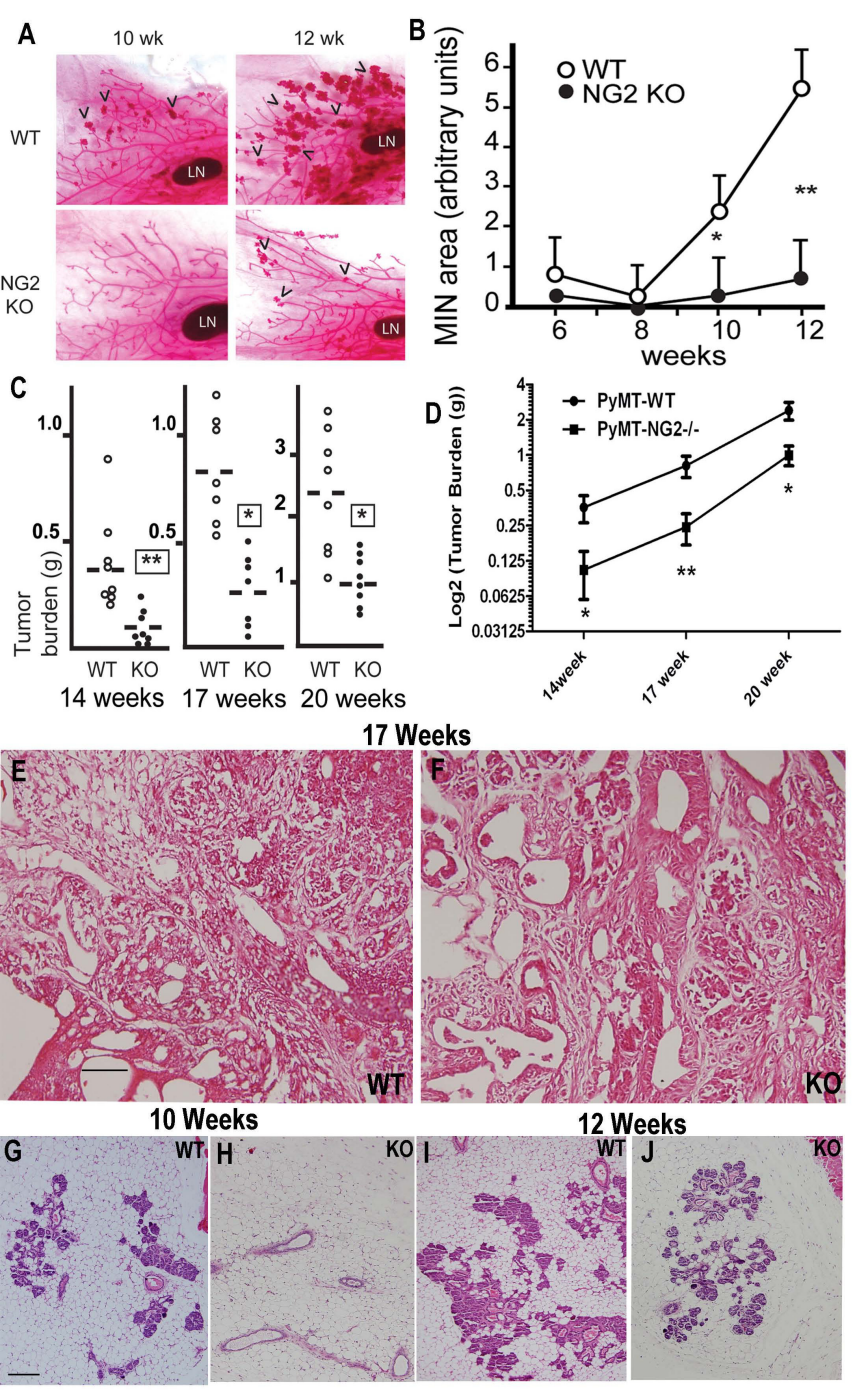
**Progression of spontaneous mammary tumors in wild type and NG2 null mice**. **A**. Whole mounts of #4 mammary glands from MMTV-PyMT female mice (WT) and NG2 null MMTV-PyMT females mice (NG2 KO) at six, eight, ten, and 12 weeks of age were stained with carmine alum to visualize the developing mammary epithelium and associated MINs. Arrowheads indicate MINs. LN: lymph node. **B**. MIN progression in wild type and NG2 null MMTV-PyMT mice was quantified by using image analysis to determine the area occupied by MINs at each time point. Large masses in the nipple area were excluded from this analysis. Eight glands were examined at each time point for each genotype. **C**. At 14, 17, and 20 weeks of age, total tumor burdens were determined in wild type and NG2 null MMTV-PyMT female mice by dissecting and weighing all tumors. Weights are in grams. At 14 weeks, *n *= 8 mice of each genotype. At 17 weeks, *n *= 7 mice of each genotype. At 20 weeks, *n *= 8 mice of each genotype. Dashed lines: average tumor weights. **D**. Semi-log plots of the data from panel C show that after an initial fast start by tumors in wild type MMTV-PyMT mice, subsequent tumor growth is similar in wild type and NG2 null hosts. * *P *= 0.03; ** *P *= 0.005. **E-F**. Sections of 17-week tumors from wild type (WT, E) and NG2 null (KO, F) MMTV-PyMT female mice were H & E stained to visualize tissue morphology. Tumors in both wild type and NG2 null hosts are multifocal, heterogeneous for cellularity and tissue morphology, and very cystic in nature. **G-J**. Sections of 10-week (G, H) and 12-week old (I, J) mammary gland from wild type (WT, G, I) and NG2 null (KO, H, J) MMTV-PyMT female mice were H & E stained to visualize MIN development. At both ages, MINs occupy larger areas in wild type than in NG2 null mammary glands. Scale bars = 100 μm in E, F and 200 μm in G-J. MIN, mammary intraepiethelial neoplasis; MMTV-PyMT, mammary tumor virus-driven polyoma middle T; NG2, nerve-glial antigen 2.

Developing MINs in the #4 mammary glands of wild type and NG2 null MMTV-PyMT female mice were examined at ages from six to 12 weeks. As previously noted, mammary tumor progression proceeds much more slowly in C57Bl/6 mice than in the more commonly used FVB strain [23]. Very little MIN development can be detected in either mouse line at six or eight weeks of age. However, at ten and 12 weeks substantial MIN development is noted in wild type mice, compared to a greatly diminished incidence of MINs in NG2 null mice (Figure 4A). Analysis of total MIN area (Figure 4B) quantifies this discrepancy in early mammary tumor development between wild type and NG2 null mice. Determinations of total tumor weights from all mammary glands were conducted to confirm the initial trend noted in the #4 mammary gland (Figure 4C). At 14 weeks, NG2 knockout mice carry only 25% of the tumor burden found in the wild type mice. At 17 weeks, the NG2 null tumor burden is about 30% of that seen in wild types. At 20 weeks, NG2 null mice still exhibit only 40% of the tumor burden found in wild type mice. When plotted in semi-log format, the data from Figure 4C reveal growth curves with roughly similar slopes for tumors in wild type and NG2 null mice (Figure 4D). Collectively, the data in Figure 4 suggest that mammary tumors in NG2 null mice have a delayed time of onset, but once established, grow at about the same rate as tumors in wild type mice. Examination of H & E-stained sections of 17-week tumors from wild type and NG2 null mice did not reveal significant differences in tumor pathology between the two genotypes (Figure 4E-F). In both wild type and NG2 null mice, these tumors are multifocal, heterogeneous with regard to cellularity and tissue morphology, and highly cystic in nature. The only reproducible difference between wild type and NG2 null specimens was the reduced number of lesions apparent at early time points in the absence of NG2 (Figure 4G-J), reinforcing the conclusions gained from the whole mount staining (Figure 4A).

#### Progression of transplanted mammary tumors

**Figure 5:**
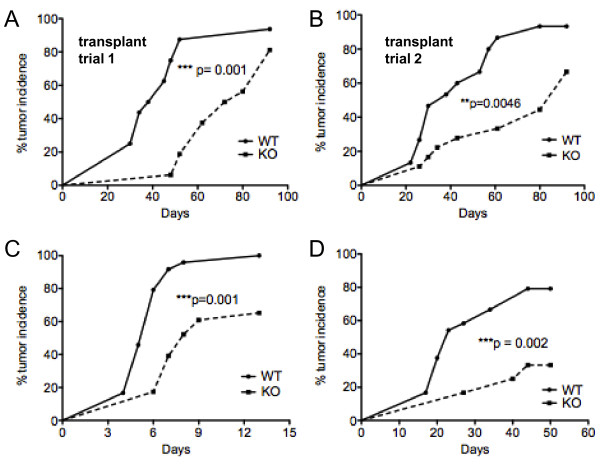
**Progression of transplanted mammary tumors in wild type and NG2 null mice**. **A, B**. 1 mm^3 ^fragments of wild type MMTV-PyMT tumors were transplanted to mammary fat pad sites in the #4 glands of wild type (wt) and NG2 null (ko) recipients. Each of the recipients (ten wild type and ten NG2 null females) received two transplants, yielding 20 potential tumor sites in each genotype. Trials 1 and 2 utilized four-month (panel A) and two-month old recipients (panel B), respectively. Data are plotted as the % sites with palpable masses as a function of time after transplantation. **C, D**. Orthotopic allografts of Py8119 (panel C) and Py230 tumor cells (panel D) were prepared by injecting 10^6 ^cells into each of four sites in the #2 and #4 mammary fat pads of wild type and NG2 null recipients (six females of each genotype; 24 potential tumor sites for each genotype). Data are plotted as the % sites with palpable masses as a function of time after transplantation. Indicated P values were obtained via Wilcoxon signed rank tests. MMTV-PyMT, mammary tumor virus-driven polyoma middle T; NG2, nerve-glial antigen 2.

Donor MMTV-PyMT tumor fragments (1 mm^3^) were transplanted into mammary fat pad sites in four-month old female wild type and NG2 null mice that did not carry the MMTV-PyMT transgene. In wild type mice, 50% of transplantation sites had detectable tumors at 40 days post-implantation. In NG2 null mice, the time for 50% incidence was extended to 80 days (Figure 5A). Similar results were obtained in a second experiment using two-month old recipient females (Figure 5B). These changes in tumor latency between wild type and NG2 null mice thus mimic the differences in latency seen with spontaneous mammary tumor development.

#### Progression of mammary tumors from cell lines

The Py230 and Py8119 cell lines were both derived from spontaneous mammary tumors growing in C57Bl/6 MMTV-PyMT mice. Both cell lines were negative for NG2 expression, as determined by immunofluorescence analysis of both cultured cells and tumors. Py8119 is negative for expression of estrogen receptor, progesterone receptor and Her2. Py230 is negative for estrogen receptor and progesterone receptor expression, but has low levels of Her2. Of the two cell lines, Py8119 has a more undifferentiated phenotype and grows more aggressively in cell culture than the more differentiated Py230. This difference is also apparent when the cell lines are grown as orthotopic tumors in the mammary fat pad. Py8119 tumors in #2 and #4 fat pads were detectable by palpation in 50% of these sites after only five days (Figure 5C), while Py230 tumors required more than 20 days to reach this stage (Figure 5D). Nevertheless, increases in tumor latency were still seen for both cell lines when injected into NG2 null mammary fat pads. For Py8119 tumors, eight days were required to reach 50% site occupancy in NG2 null mice, while this 50% level was never achieved in the case of Py230 tumors.

### Vascularization of mammary tumors in wild type and NG2 null mice

#### Vascularization of spontaneous tumors

**Figure 6:**
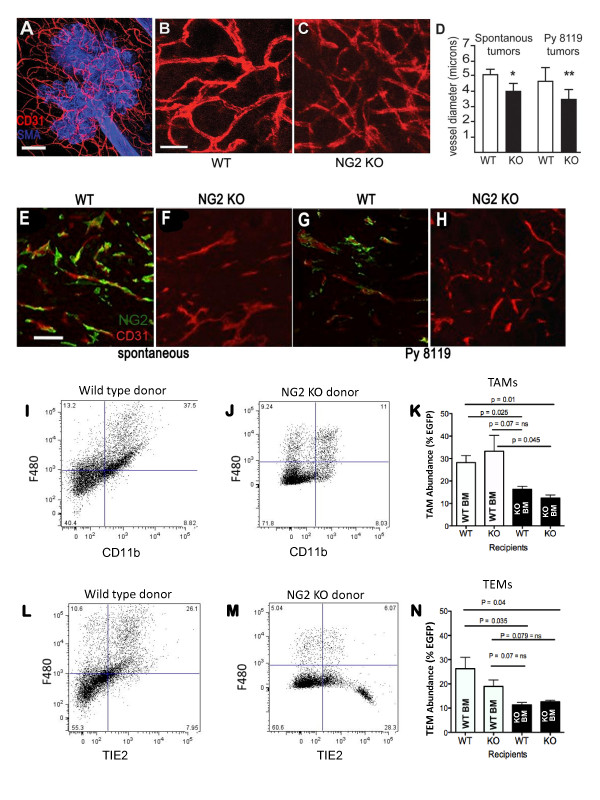
**Vessel diameter and macrophage phenotype in wild type and NG2 null mice**. **A-C**. Whole mounts of #4 mammary glands from 14-week old wild type (WT) and NG2 null (KO) MMTV-PyMT females were immunostained for CD31 (red) and αSMA (blue) to investigate vascularization of incipient neoplasms. Although not shown in B and C, αSMA staining was used as illustrated in A to restrict analysis of CD31-positive vessels to areas within developing MINs. Thus, we only analyzed tumor-associated vessel segments and not vessels associated with normal areas of the mammary gland. Tumor-associated vessels in wild type mice (B) appear thicker and are more robustly stained for CD31 than vessels in NG2 null mice (C). To quantify vessel size, ten vessel diameters were measured in each of five fields from each of 12 MINs for each genotype to yield the data plotted in the left-hand side of panel **D **(spontaneous tumors). Sections of CD31-immunolabeled Py8119 fat pad tumors (at day 12) were used to obtain the data shown on the right-hand side of panel D (Py8119 tumors). A total of 20 vessel diameters were determined in each of six sections from each of four 12-day tumors in each genotype. Scale bars = 100 μm in A and 25 μm in B. * *P *= 0.02; ** *P *= 0.007. Double immunostaining for CD31 (red) and NG2 (green) was performed on sections of 17-week spontaneous tumors from wild type (**E**) and NG2 null (**F**) MMTV-PyMT females and on sections of 12-day Py8119 tumors from wild type (**G**) and NG2 null (**H**) female mice. In both types of tumors, NG2 labeling is seen in association with CD31-positive endothelial cells in wild type hosts, but not in NG2 null hosts. Bar in E = 25 μm. TAM abundance (**I-K**) and TEM abundance (**L-N**) were studied by flow cytometric analysis of tumor macrophages in wild type and NG2 null recipients transplanted with wild type or NG2 null β-actin/EGFP bone marrow. F4/80, CD11b, CD45, and Gr1 were used for analysis of TAMs, while F4/80, Tie2, CD206, and CD11c were used for analysis of TEMs. Analysis was restricted to EGFP-positive cells. FACS plots I-J show only the F4/80 and CD11b pairing, while plots L-M show only the F4/80 and Tie2 pairing. In addition, only NG2 null recipient data are shown. Numbers in each quadrant of the FACS plots indicate the percentage of the total number of EGFP-positive cells analyzed. Data for wild type recipients appear very similar. Abundance of TAMs and TEMs are indicated in panels K and N, respectively, as percentages of the total number of EGFP-positive cells. Open bars = wild type bone marrow donors. Black bars = NG2 null bone marrow donors. These data are based on the use of all four markers, not just the two shown in the FACS plot examples. All values in panels K and N are derived from FACS analysis of five separate tumors, all from different mice. Three of the eight *P *values approach, but do not reach, a statistical significance level of 0.05. EGFP, enhanced green fluorescent protein; FACS, fluorescence activated cell sorting; MMTV-PyMT, mammary tumor virus-driven polyoma middle T; NG2, nerve-glial antigen 2; αSMA, α smooth muscle actin.

In this initial report we have focused on the vascular role of NG2 in mammary tumorigenesis. Whole mounts of 14-week mammary glands from wild type and NG2 null MMTV-PyMT females were double immunolabeled with antibody against CD31 to visualize the vasculature and with antibody against αSMA to visualize mammary ducts and MINs budding from these ducts. Figure 6A shows the CD31-positive vascular network present within an αSMA-positive MIN in the wild type mouse. Viewed at high magnification, tumor-associated vessels in NG2 null tumors (Figure 6C) appear to be thinner and somewhat less robustly labeled for CD31 compared to those seen in wild type tumors (Figure 6B). Quantification of vessel diameter in a panel of 12 MINs from each genotype confirms a statistically significant 20% difference between vessels in wild type and NG2 null tumors (Figure 6D). Figure 6E-F confirms the absence of NG2 expression in the stroma of spontaneous mammary tumors in NG2 null MMTV-PyMT mice.

#### Macrophage phenotypes in spontaneous tumors

Although the bulk of our analysis of tumor vasculature focuses on NG2-dependent changes in pericyte function, myeloid cells can also make important contributions to tumor vascularization [26-28]. We have therefore made an initial study of the effects of NG2 ablation on myeloid cell populations in spontaneous MMTV-PyMT tumors. These studies were conducted with gamma-irradiated MMTV-PyMT female mice engrafted with bone marrow from wild type β-actin/EGFP donors and from NG2 null β -actin/EGFP donors, allowing us to control the genotype of macrophages recruited to the mammary tumors. Use of the EGFP reporter further allowed us to restrict our phenotypic analysis to macrophages derived from the bone marrow, as opposed to tissue resident macrophages. Flow cytometric analysis of dissociated mammary tumors at 16 to 18 weeks of age reveals that transplantation of NG2 null bone marrow, regardless of the recipient genotype, results in reduced numbers of tumor-associated macrophages (TAMs) characterized by the marker phenotype F4/80-positive, CD11b-positive, CD45-positive, Gr1-negative (Figure 6I-K). The FACS plots in Figure 6I-J show representative data only for the F4/80 and CD11b markers and only for NG2 null recipients. FACS data for wild type recipients are very similar (not shown for space considerations). Quantification of macrophage abundance in Figure 6K is based on the use of all four markers. Similarly, transplantation of NG2 null bone marrow, regardless of recipient genotype, reduces the number of Tie2-expressing macrophages (TEMs) characterized by the marker phenotype F4/80-positive, Tie2-positive, CD206-positive, CD11c-positive (Figure 6L-N). Again, the FACS plots in Figure 6L-M show representative data only for the F4/80 and Tie2 markers and only for NG2 null recipients. FACS data for wild type recipients are very similar. Quantification in Figure 6N is based on the use of all four markers. These data suggest that NG2 may be important for the recruitment of myeloid cells and/or the maturation of myeloid cells to TAMs and TEMs, both of which are thought to have tumor promoting properties. Future studies will need to address more directly the impact of NG2 ablation on macrophage involvement in mammary tumor progression itself, as well as in vascularization and other aspects of mammary tumorigenesis.

#### Vascularization of orthotopic allograft tumors

Although we have developed a panel of assays for examining vessel structure and function [16], the heterogeneous, multifocal, and non-synchronous nature of spontaneous tumors in the MMTV-PyMT model [5] frustrated our attempts to obtain reproducible data concerning these vascular parameters. We therefore turned to analysis of tumors produced by the mammary tumor cell lines, which are monofocal and can be grown in relatively synchronous fashion in cohorts of animals.

Vascularization parameters were examined in orthotopic fat pad tumors derived from the Py8119 cell line. We focused on relatively early stages of tumor development for two reasons. First, the types of morphological and functional analyses we wanted to perform are very difficult to carry out satisfactorily in later-stage tumors that are characterized by highly abnormal vessels and large areas of necrosis. Second, based on previous experience [15,16], we felt that ablation of NG2 was likely to affect the timing of the angiogenic switch, the initial establishment of a functional tumor vasculature that is critical for the early success of tumor survival and progression. Thus, for each type of vascular analysis described here, tumors of similar sizes (2 to 3 mm diameter; average time of development nine days for tumors in wild type mice, 12 days for tumors in NG2 null mice) from wild type and NG2 null hosts were chosen for study. Vessel diameter (determined from CD31-stained sections) was reduced by 20% in tumors from NG2 null mice (Figure 6D), confirming the similar difference in vessel diameter seen in spontaneous tumors. Figure 6G-H confirms the absence of NG2 expression in Py8119 tumors growing in NG2 null mice.

**Figure 7:**
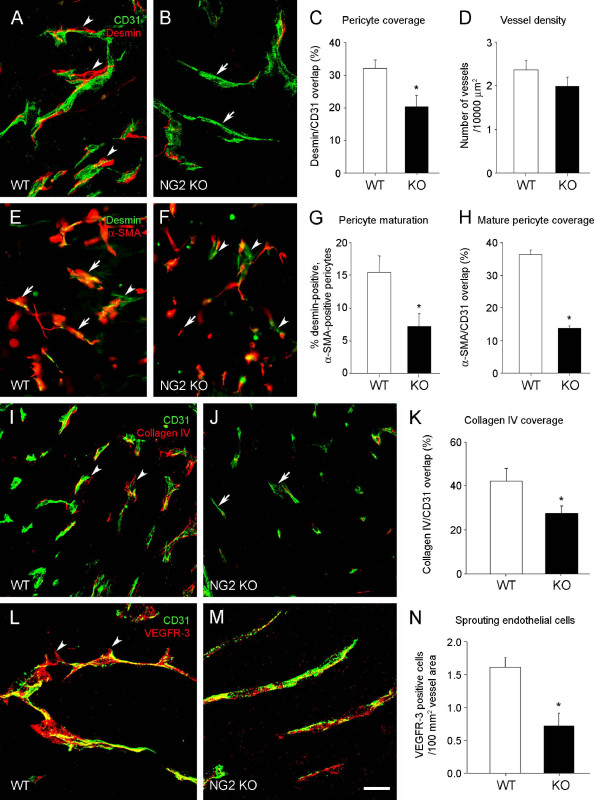
**Structural deficits of vessels in mammary tumors in NG2 null mice**. Py8119 fat pad tumors (2-3 mm diameter) in wild type (WT) and NG2 null (NG2 KO) recipients were used to evaluate several types of structural deficits in tumor vessels due to ablation of NG2. **A-C**. Pericyte coverage of endothelial cells was evaluated in wild type (A) and NG2 null (B) tissue sections immunostained for desmin (red) and CD31 (green). Since desmin and CD31 are on distinct cell types, they cannot overlap in a single optical section. However, because of the intimate interaction between pericytes and endothelial cells, the two labels appear to overlap when viewed in three-dimensional space. Analysis of confocal z-stacks therefore allows quantification of the extent to which desmin appears to overlap with CD31 labeling. Arrowheads in A indicate areas of overlap between desmin and CD31. Arrows in B show vessel segments without pericyte coverage. The extent to which CD31 pixels are overlapped by desmin pixels provides a measure of pericyte ensheathment of endothelial cells (C). Data were collected from eight tumors per genotype, evaluating four sections per tumor. **D**. Vascular densities are not significantly different in Py8119 tumors in wild type and NG2 null mice, as quantified by counting CD31-positive vessels in a 10,000 μm^2 ^area. Data were collected from eight tumors per genotype, evaluating four sections per tumor. **E-G**. Pericyte maturation was evaluated via double immunostaining for desmin (green, all pericytes) and αSMA (red, mature pericytes). Mature pericytes express both desmin and αSMA (arrows), while immature pericytes express only desmin (arrowheads). Pericyte maturation is calculated as the % of desmin-positive pericytes that are αSMA-positive (G). Data were collected from four tumors per genotype, evaluating three sections per tumor. **H**. Endothelial ensheathment by mature pericytes was quantified by double immunostaining for CD31 and αSMA. Because the number of mature pericytes is reduced in tumor vessels in NG2 null hosts, only vessels with αSMA-positive pericytes are included in this analysis. Endothelial investment by mature pericytes is quantified as % overlap of CD31 pixels by αSMA pixels. For each genotype, five selected vessels each were examined in three different sections from each of three tumors. **I-K**. Basal lamina assembly was evaluated by immunostaining for CD31 (green) and collagen IV (red) in wild type (I) and NG2 null (J) tumor sections. Arrowheads in I indicate areas of collagen IV/CD31 overlap. Arrows in J show vessel segments with poor basal lamina deposition. Confocal Z-stacks were used to determine the percentage of CD31-positive pixels covered by collagen IV pixels (K). Data were collected from six tumors per genotype, evaluating four sections per tumor. **L-M**. Endothelial cell sprouting was evaluated by double immunostaining for CD31 (green) and VEGFR-3 (red) in wild type (L) and NG2 null (M) tissue sections. Arrowheads in M indicate VEGFR-3 high/CD31-low structures that characterize sprouting endothelial cells. Confocal z-stacks were used to determine the number of sprouting tip cells per 100 mm^2 ^of CD31-positive vessel area (**N**). Data were collected from four tumors per genotype, evaluating three sections per tumor. Scale bars = 20 μm (A, B), 90 μm (E, F), 30 μm (I, J), and 12 μm (L, M). * *P *= 0.02; ** *P *= 0.006. NG2, nerve-glial antigen 2; αSMA, α-smooth muscle actin; VEGFR-3, vascular endothelial growth factor receptor-3.

Since NG2 ablation disrupts pericyte recruitment [15] and pericyte/endothelial cell interaction [16], we next used pericyte-specific (desmin) and endothelial-specific (CD31) markers for confocal microscopic evaluation of the extent of pericyte ensheathment (coverage) of endothelial cells. By quantifying the percentage of CD31 pixels that are overlapped by desmin pixels, Figure 7A-B illustrates the relative decrease in desmin-positive pericyte association with CD31-positive endothelial cells in the NG2 null mouse. Quantification of pericyte coverage of endothelial cells reveals a 35% decrease in the case of NG2 null tumor vessels (Figure 7C). This change in pericyte ensheathment in the NG2 null mouse did not significantly affect vascular density, as determined by counting the number of CD31-labeled tumor vessels per unit area in tumors from both genotypes (Figure 7D).

Altered pericyte/endothelial cell interaction due to NG2 ablation is accompanied by decreased pericyte maturation, as revealed by double-staining for desmin and the mature pericyte marker αSMA (Figure 7E-F). While desmin-positive, αSMA-positive pericytes are present in tumor vessels in both wild type and NG2 null mice (arrows), the abundance of these mature cells is reduced two-fold in the absence of NG2 (Figure 7G). Desmin-positive, αSMA-negative cells (arrowheads) are correspondingly more abundant in tumor vessels in NG2 null mice. Since the maturation of pericytes could have an effect on their ability to ensheath endothelial cells, we also used double labeling for αSMA and CD31 to determine whether endothelial cell investment by mature pericytes is still deficient in tumor vessels in NG2 null mice. These measurements show that, relative to the situation in wild type tumors, coverage of endothelial cells by αSMA-positive mature pericytes is reduced three-fold in tumor vessels in the NG2 null mouse (Figure 7H). The absence of NG2 thus has negative effects on both pericyte maturation and pericyte investment of endothelial cells.

We used a similar type of pixel overlap strategy in conjunction with CD31/collagen IV double labeling to compare vascular basal lamina assembly in Py8119 tumors grown in wild type and NG2 null hosts (Figure 7I-J). These results reveal a 50% deficit in collagen IV association with blood vessels in mammary tumors from NG2 null mice (Figure 7K), indicative of reduced basal lamina assembly as a consequence of subnormal pericyte/endothelial cell interaction.

To examine whether diminished pericyte/endothelial interaction and basal lamina assembly affect the development of vascular endothelial cells, we utilized vascular endothelial growth factor receptor 3 (VEGFR-3) as a marker expressed in the filopodia of sprouting endothelial tip cells [29]. Double immunostaining for VEGFR-3 and CD31 demonstrates abundant VEGFR-3 expression in vessels of Py8119 mammary tumors growing in wild type mice, but significantly less VEGFR-3 expression in vessels of tumors in NG2 null mice (Figure 7L-M). Quantification of VEGFR-3 expression relative to vessel area reveals a two-fold decrease in endothelial cell sprouting in tumor vessels in the NG2 null mouse, compared with tumor vessels in the wild type mouse (Figure 7N).

**Figure 8:**
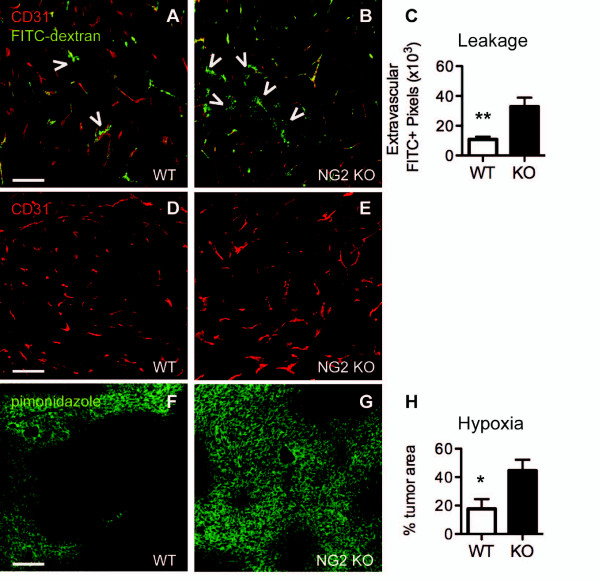
**Deficits in vessel function in mammary tumors in NG2 null mice**. A-C. Leakage from Py8119 tumor vessels was evaluated using intravenously-administered FITC-dextran. Tissue sections were immunolabeled for CD31 (red) to allow image analysis of FITC-dextran (green) located external to tumor vessels in wild type (A) versus NG2 null (B) tissues. Arrowheads in merged images A and B show sites with extravascular FITC-dextran. Confocal z-stacks were used to quantify the percentage of FITC pixels external to CD31-positive vessel walls (C). Data were collected from six tumors per genotype, evaluating four sections per tumor. **D-H**. Tumor hypoxia was evaluated in mice injected intravenously with pimonidazole hypoxia probe. Immunostaining of wild type (D, F) and NG2 null (E, G) tissue sections for CD31 (red, D, E) and pimonidazole (green, F, G) allowed visualization of hypoxic areas relative to tumor vasculature. Image analysis was used to determine the extent of hypoxia as a percentage of total tumor area (H). Data were collected from six tumors per genotype, evaluating four sections per tumor. Scale bars = 100 μm. * *P *= 0.05; ** *P *= 0.003. FITC, fluorescein isothiocyanate; NG2, nerve-glial antigen 2.

Impaired pericyte/endothelial cell interaction, reduced basal lamina assembly, reduced pericyte maturation and altered endothelial cell biology in the NG2 null mouse have important consequences for tumor vessel function. Figure 8A shows that intravenously-injected FITC-dextran is largely retained within the boundaries of CD31-positive tumor vessels in the wild type mouse. In contrast, a significant amount of FITC-dextran is found external to tumor vessels in the NG2 null mouse (Figure 8B). Quantification of extravascular FITC-dextran reveals more than a three-fold increase in vessel leakiness in NG2 null tumor vessels (Figure 8C). Impaired vessel function also leads to elevated tumor hypoxia in NG2 null mice (Figure 8E, G), to a much greater extent than in wild type mice (Figure 8D, F) as shown by use of a pimonidazole hypoxia probe. Quantification of pimonidazole-positive area indicates a more than two-fold increase in hypoxia in tumors from NG2 null mice, relative to that seen in tumors in wild type mice (Figure 8H).

**Figure 9:**
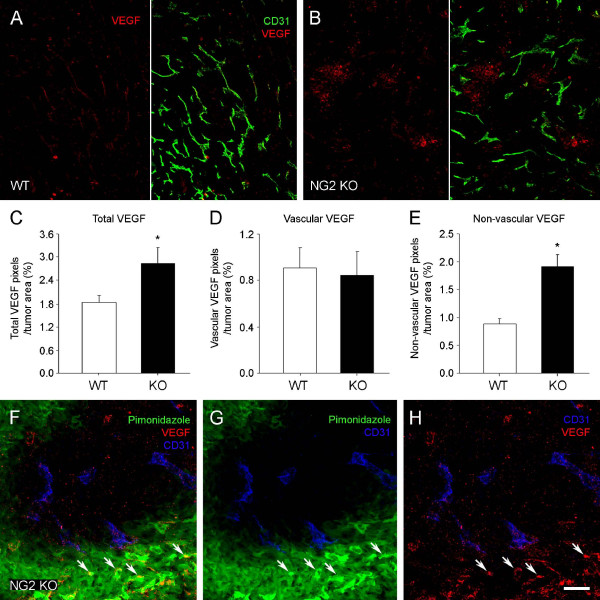
**Increased VEGF expression in mammary tumors in NG2 null mice**. A, B. Because hypoxia induces VEGF expression, we used double immunostaining for VEGF (green) and CD31 (red) to localize VEGF expression relative to tumor blood vessels in wild type (WT, A) and NG2 null (NG2 KO, B) hosts. Some VEGF is associated with CD31-positive tumor blood vessels and some VEGF is dispersed in the tumor tissue. Evaluating overlap of VEGF and CD31 pixels allowed quantification of these two different pools of VEGF. Total VEGF is increased in tumors from NG2 null hosts (**C**). This VEGF increase in NG2 null tumors is due to non-vascular VEGF dispersed in the tumor tissue (**E**), rather than to VEGF closely associated with vessels (**D**). Data were collected from four tumors per genotype, evaluating four sections per tumor. **F-H**. Triple immunostaining for VEGF, CD31, and pimonidazole hypoxia probe in NG2 null tumors shows that non-vascular VEGF (arrows) is localized to hypoxic areas lacking blood vessels. Scale bars = 60 μm (A, B) and 20 μm (F-H). NG2, nerve-glial antigen 2; VEGF, vascular endothelial growth factor.

Since hypoxia is known to induce expression of the angiogenic growth factor VEGF, accompanied by vascular remodeling [30], we examined Py8119 tumors in wild type and NG2 null hosts to determine if VEGF expression was affected by the elevated hypoxia levels seen in NG2 null tumors. Immunostaining for VEGF reveals detectable levels of the growth factor in 12-day tumors in both types of hosts (Figure 9A-B). The double labeling for VEGF and CD31 shows that some of this VEGF is associated with tumor blood vessels, while the rest has a non-vascular distribution in the tumor tissue. The total amount of VEGF is higher in NG2 null tumors than in wild type tumors (Figure 9C). However, quantification of VEGF pixels that overlap with CD31 pixels reveals that vessel-associated VEGF levels are similar in wild type and NG2 null tumors (Figure 9D), while non-vascular VEGF accounts for the increased level of growth factor seen in NG2 null tumors (Figure 9E). Figures 9 F-H demonstrate that this non-vascular VEGF in NG2 null tumors (arrows) is localized to pimonidazole-labeled hypoxic areas lacking CD31-positive blood vessels. In spite of the increased VEGF levels found in NG2 null tumors, vascular density remains similar to that found in wild type tumors (Figure 7D), at least at this early time point.

## Discussion

Previous work has shown that the NG2 proteoglycan promotes cell proliferation and motility, along with cell-cell and cell-matrix interactions [13]. Mechanisms underlying these effects involve the ability of NG2 to potentiate cellular responses to growth factors [13,31] and to activate signaling by β1 integrins [32,33]. These functions of NG2 make it an important player in pericyte biology, as evidenced by our findings that genetic ablation of the proteoglycan leads to vascularization deficits in both tumor [16,34,35] and non-tumor [15] models. However, the detailed effects of NG2 ablation on vascular structure and function have been examined only in the case of intracranial melanoma allografts [16]. Thus, the importance of vascular NG2 in other types of tumors outside the brain, especially spontaneous tumors, remains unexplored. In this report we examine effects of NG2 ablation on the cellular and functional properties of tumor vessels in mammary tumors.

In three different experimental paradigms (orthotopic tumor cell line allografts, tumor transplantation, and *de novo *tumor development), the appearance of detectable MMTV-PyMT mammary tumors is significantly delayed in the NG2 null mouse. The normal pattern of mammary gland development seen in the NG2 null mouse suggests that impaired mammary tumorigenesis is not the result of NG2-dependent deficits in normal mammary morphogenesis. Instead, the role of NG2 manifests itself during the process of tumorigenesis. In the case of melanomas and gliomas, NG2 is highly expressed by components of the tumor stroma [13,36,37] but can also contribute to tumor progression as a component of the tumor cells themselves [32,38]. In human breast cancer, NG2 is also reported to promote tumor progression via its expression on so-called triple negative tumor cells [18,19]. However, in the case of MMTV-PyMT mammary tumors, our immunocytochemical studies establish that NG2 is not expressed by either the mammary epithelium or by neoplastic mammary tumor cells derived from this normal tissue. Instead, the proteoglycan is found on adipocytes in the mammary fat pad, myeloid cells that invade tumors from the circulation, and on perivascular cells associated with tumor microvessels. We see this same pattern of NG2 expression in samples of non-triple negative human ductal adenocarcinoma. The tumor-promoting properties of stromal NG2 further attest to the powerful influence of stromal elements on mammary tumor progression. These stromal effects of NG2 will be important for both mammary and other types of tumors, regardless of whether the tumor cells themselves are NG2-positive or NG2-negative.

Significantly, increased tumor latency is the most apparent effect of NG2 ablation in all three paradigms that we tested (orthotopic allografts, transplants, and *de novo *tumors). While tumor onset is delayed in the NG2 null mouse in each of these models, once tumor growth begins, it occurs at roughly the same rate observed in wild type mice. These observations seem compatible with the evidence we present concerning the importance of NG2 for effective early tumor vascularization, and also with the idea that early establishment of a functional vascular supply is a critical event in the successful progression of a tumor. Since robust tumor vascularization is required for the angiogenic switch from hyperplasia to neoplasia [7,8,39,40], for continued tumor growth, and for eventual dispersal of tumor cells to distant sites, factors that reduce vascularization frequently are inhibitory to tumor progression. The retarded early progression of mammary tumor growth we have observed in the NG2 null mouse is consistent with the impaired progression in this mouse of other vascularization-based pathologies [15], including brain tumor progression [16].

The fact that NG2, an early marker of activated pericytes, plays an important functional role in vascularization is a testament to the precocious role of pericytes in the neovascularization process. As opposed to the traditional view of pericytes as late participants in the vascularization process, more recent studies have demonstrated the early presence of these cells in nascent microvessels [9,10,12,25,41-43], especially in the context of tumor vascularization [11,16]. Several pieces of our current evidence point to differences in early vascularization of mammary tumors in wild type and NG2 null mice. As observed in pathological ocular neovascularization [15] and brain tumor vascularization [16], NG2 null pericytes exhibit reduced ensheathment of endothelial cells in mammary tumors. Although a reduction in pericyte number is not apparent in tumors growing in NG2 null mice [16], reduced pericyte interaction with endothelial cells nevertheless compromises pericyte contribution to vessel development. This suggests that a key role of pericyte NG2 is the mediation of pericyte/endothelial cell communication via stimulation of β1 integrin signaling in endothelial cells [17]. This communication deficit is also reflected by reduced assembly of the vascular basement membrane in mammary tumors grown in the NG2 null mouse. Deposition of the vascular basal lamina is a key result of pericyte/endothelial cell collaboration, since this structure is critical for vessel maturation, maintenance, and function [44,45].

The reductions in both pericyte coverage of endothelial cells and basal lamina deposition in mammary tumor vessels in the NG2 null mouse are accompanied by several other deficits in vessel structure and function. Vessels in NG2 null tumors are smaller in diameter than those in wild type tumors. The 20% reduction in vessel diameter seen in both spontaneous and engrafted tumors might restrict blood flow to tumor tissue in the NG2 null mouse. Pericyte maturation is retarded in tumor vessels in the NG2 null mouse and endothelial cell investment by mature pericytes is impaired to a greater extent than investment by immature pericytes. Endothelial cell sprouting is also reduced in tumor vessels in the NG2 null mouse, consistent with numerous studies demonstrating the importance of extracellular matrix attachment for activation of key signaling pathways in endothelial cells [46-48]. As a result of these multiple defects, early tumor vessels in the NG2 null mouse exhibit greater than three-fold increased leakiness compared to early vessels in wild type tumors. The overall consequence of these deficits is an almost three-fold increase in mammary tumor hypoxia in the NG2 null mouse. All of these vascular deficiencies are observed during the same early time period in which tumor establishment and growth are negatively impacted in the NG2 null mouse.

Elevated hypoxia in early-stage NG2 null tumors is accompanied by expression of increased levels of VEGF beyond what is seen in wild type tumors. Levels of vessel-associated VEGF are similar in tumors in both genotypes, so that increased amounts of non-vascular VEGF account for the difference in VEGF levels seen in wild type and NG2 null tumors. This VEGF localization pattern may account for our observation that increased VEGF levels in NG2 null tumors are not accompanied by changes in vascular density or morphology. Our previous study with tumors in collagen VI null mice suggested that tumor vessel remodeling is more readily induced by vessel-associated VEGF [49]. Extracellular matrix-bound VEGF species have been shown to have angiogenic/morphogenetic properties that differ from those of non-vascular VEGF species [47]. Increased levels of non-vascular VEGF are apparently not sufficient to induce vascular growth and remodeling, at least at this early stage of tumor development. It remains to be determined whether increased VEGF levels in NG2 null tumors can have effects on vascular density and/or morphology at later points in tumor development. It will also be important to investigate the long-term effects of NG2 ablation on tumor vascularization and hypoxia, with specific attention to tumor growth, invasion, and metastasis. An important feature of the MMTV-PyMT mouse mammary tumor model, metastasis is a critical factor in determining the survival of human breast cancer patients.

Reduced mammary tumor progression in the MMTV-PyMT mouse due to pericyte-dependent deficits in vascularization has a parallel in endothelial cell-dependent deficits in the tumor vasculature. In the context of the MMTV-PyMT model, ablation of T-cadherin, which is normally expressed by vascular endothelial cells, causes deficiencies in mammary tumor vascularization that lead to diminished tumor progression [50]. In addition to highlighting the importance of pericyte/endothelial cell crosstalk, these combined findings once again emphasize the critical dependence of mammary tumor progression on vascularization. The interplay between pericytes and endothelial cells validates recent attempts at dual targeting of these two cell types as a means of improving the efficacy of anti-angiogenic therapy [51,52]. A dual targeting strategy might offer the means of improving therapy in cases of breast cancer that are resistant to other types of treatment.

In spite of our evidence that ablation of pericyte NG2 is an important factor in the reduced mammary tumor progression seen in NG2 null mice, we must still confront the possibility that ablation of NG2 in myeloid cells and adipocytes may also contribute to the observed effects. The frequent perivascular localization of myeloid cells [12,27], along with the emerging importance of these cells in several aspects of tumor progression, including inflammation, vascularization [26,28,40,53], and metastasis [54-56], demands that we consider the effects of NG2 ablation on myeloid cell function. Our own work has demonstrated the participation of NG2-positive myeloid cells in the earliest stages of fibroblast growth factor 2 (FGF2)-induced neovascularization [12]. Our initial flow cytometry evidence indicates that NG2 ablation reduces the number of both TAMs and TEMs present in mammary tumors. Both of these macrophage sub-populations are thought to have tumor promoting properties [26-28,53-56], consistent with our observation of delayed mammary tumor growth in NG2 null MMTV-PyMT mice. Along these same lines, we have previously seen that ablation of NG2 in a model of spinal cord demyelination diminishes macrophage recruitment to demyelinated lesions and shifts macrophages from a pro-inflammatory to anti-inflammatory phenotype [57]. These apparent effects of NG2 on macrophage recruitment and/or maturation emphasize the need for additional work to determine the role of the proteoglycan in macrophage contributions to tumor vascularization, growth and metastasis. We cannot conclude from our current results whether changes in macrophage populations are merely correlated with changes in tumor growth or whether they are causally involved in altering tumor growth.

Adipocytes also have the potential to play a key stromal role in mammary tumorigenesis. New discoveries are defining the role of adipocytes in controlling metabolism, as well as in producing adipokines that promote mammary tumor progression [58,59]. It is of considerable interest that ablation of the NG2 ligand collagen VI, which is a product of adipocytes, leads to impaired mammary tumor progression in the MMTV-PyMT mouse [60]. Since NG2 and collagen VI are both produced by adipocytes, and since NG2 serves as a cell surface receptor for collagen VI [61,62], NG2 on the adipocyte surface might be important for collagen VI anchorage and localization, and perhaps for its effects on the behavior of mammary tumor cells.

In order to resolve these questions regarding the various stromal roles of NG2 in breast cancer, we are developing Cre-lox capabilities for cell type-specific ablation of the proteoglycan in the context of the MMTV-PyMT model. This will allow us to define the importance of NG2 in mediating the respective effects of pericytes, myeloid cells, and adipocytes on mammary tumor progression. Understanding the respective roles of NG2 in these different stromal populations will be important for any attempts to use the proteoglycan as a target for therapeutic purposes. For cases of human breast cancer in which the tumor cells also express NG2, targeting of the proteoglycan may be an even more powerful approach due to inhibitory effects on both tumor and stromal compartments.

## Conclusions

In mammary tumors in the MMTV-PyMT transgenic mouse, the NG2 proteoglycan is not expressed by the mammary tumor cells, but is expressed by at least three components of the tumor stroma: microvascular pericytes, myeloid cells, and adipocytes. Global ablation of NG2 in this system greatly slows the early growth of spontaneous, transplanted and allografted mammary tumors, demonstrating the powerful effect of stromal elements on mammary tumor progression. Due to the role of NG2 in pericyte biology, pericyte ensheathment of endothelial cells is diminished during the early stages of tumor vascularization in NG2 null mice, accompanied by reduced pericyte maturation, reduced sprouting of endothelial cells, diminished assembly of the vascular basal lamina, and smaller vessel diameter. As a result of these early changes in vascular development, tumor vessels exhibit increased leakiness in the NG2 null mouse and tumor hypoxia is greatly increased. These vascular deficits correlate with the early decreases in tumor growth seen in the absence of NG2, suggesting that NG2-dependent vessel development and function are important for the angiogenic switch that precedes subsequent neoplastic progression. Initial studies of macrophage phenotypes in NG2 null mice suggest that NG2 ablation may also compromise tumor macrophage function, possibly including macrophage contributions to tumor vascularization. Cell type-specific ablations of NG2 in individual stromal compartments will be required to dissect the respective roles of the proteoglycan in pericyte and macrophage function.

## Abbreviations

BSA: bovine serum albumin; EGFP: enhanced green fluorescent protein; FACS: fluorescence activated cell sorting; FCS: fetal calf serum; FGF2: fibroblast growth factor-2; FITC: fluorescein isothiocyanate; H & E: hematoxylin and eosin; LN: lymph node; MIN: mammary intraepithelial neoplasia; MMTV-PyMT: mammary tumor virus-driven polyoma middle T; NG2: nerve-glial antigen 2; PBS:: phosphate-buffered saline; SE: standard error; αSMA: α-smooth muscle actin; VEGF: vascular endothelial growth factor; VEGFR-3: vascular endothelial growth factor receptor-3.

## Competing interests

The authors declare that they have no competing interests.

## Authors' contributions

KG and WKY contributed equally to this work, participating in study design, data acquisition and analysis, and preparation of the manuscript. KK, HH, LJTY, and YC performed data acquisition and analysis. LGE contributed key resources and participated in manuscript preparation. RDC and WBS contributed to experimental design and to preparation of the manuscript. All authors read and approved the final manuscript.
